# Phase-channel dynamics reveal the role of impurities and screening in a quasi-one-dimensional charge-density wave system

**DOI:** 10.1038/s41598-017-02198-x

**Published:** 2017-05-17

**Authors:** M. D. Thomson, K. Rabia, F. Meng, M. Bykov, S. van Smaalen, H. G. Roskos

**Affiliations:** 1 Physikalisches Institut, J. W. Goethe-Universität, 60438 Frankfurt am Main, Germany; 2 Laboratory of Crystallography, University of Bayreuth, 95440 Bayreuth, Germany

## Abstract

Charge density waves (CDWs), i.e. the periodic spatial modulation of coupled electronic and lattice density, are ubiquitous in low-dimensional conductors and have taken on renewed relevance due their role in state-of-the-art materials, e.g. high-*T*_*c*_ superconductors, topological insulators and low-dimensional carbon. As CDWs are described by a complex order parameter to represent both the amplitude and phase, they are formally analogous to BCS superconductors and spin-waves, providing a prototype of collective phenomena for the further development of field theories and ab-initio calculations of complex solids. The low-energy excitations are mixed electron-phonon quanta which ideally separate into an amplitude and phase channel, and provide a sensitive probe of the ground state and non-equilibrium dynamics, including ultrafast photoinduced phase transitions. While recent studies of the amplitude modes have brought substantial progress aided by a phenomenological Ginzburg-Landau framework, we focus here on the phase modes using ultrafast terahertz spectroscopy. Experiments on K_0.3_MoO_3_ provide a more complete picture, and reveal a high sensitivity to interactions with impurities and screening effects from photogenerated carriers, both of which can be accounted for by generalizations of the model. Moreover, our considerations emphasize the need to revisit the treatment of inherent electronic damping in quantum-mechanical CDW theories.

## Introduction

**Figure 1:**
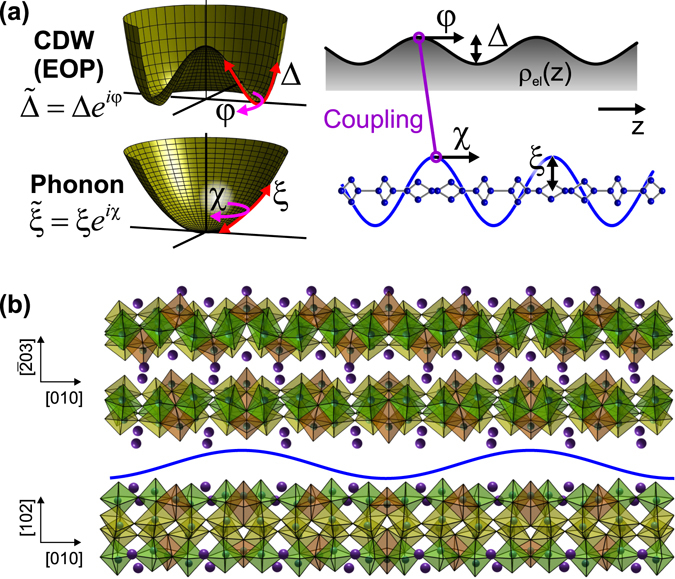
Representation of quasi-1D charge density wave and structure in K_0.3_MoO_3_. (**a**) Schematic of phase-space potentials (left) and spatial modulation (right) associated with electronic CDW (order parameter, EOP) and a single phonon, with wavevectors *q* = 2*k*_F_ and complex amplitudes $\tilde{{\rm{\Delta }}}$ and $\tilde{\xi }$, respectively. (**b**) Structure of K_0.3_MoO_3_ (*T* = 100 K^43^) including CDW distortion (exaggerated by a factor 20 for clarity), assuming **q** ≈ [0,0.75,0.5]. Crystal is truncated by the experimental ($20\bar{1}$) sample surface plane (containing [010] and [102] directions); Mo-O octahedra color-coded by inequivalent Mo atoms in the undistorted *C*2/*m* lattice.

The low-temperature CDW phase in organic and inorganic solids^1^ serves as an important prototype for a broad range of collective phenomena involving strongly coupled degrees of freedom and spontaneously symmetry-broken ground states^2^. Recently, the role of CDWs in low-dimensional systems has become more generally recognized, as they are found to arise in high-*T*_c_ superconductors^3^ and topological insulators^4^ as competing excitations, as well as in graphene- and carbon-nanotube-based materials^5, 6^. The low-energy excitations of the incommensurate CDW ground state (GS) correspond to distortions of the coupled electron and lattice density modulation (at *q* = 2*k*_F_), which ideally decouple into a (Raman-active) amplitude channel and (infrared-active, Goldstone) phase channel, and can be exploited as sensitive spectroscopic probes of the CDW physics. Although many studies focused on a single coupled phonon^1, 7^, which leads to an “amplitudon” and “phason” (the latter a soft mode at frequency *ν* ~ 0), in general multiple phonons can couple to the CDW electrons yielding a set of amplitude- and phase-“phonons”^8–12^. While quantum-mechanical (QM) descriptions were developed at an early stage^8, 9^ which predict such modes (based on fluctuations of the Fröhlich Hamiltonian^7^), recently a phenomenological time-dependent Ginzburg-Landau (TDGL) model was employed to account for both the temperature dependence of the modes and non-equilibrium dynamics upon photoexcitation^11–13^. The model describes the *Q* = 0 distortions of the CDW via classical equations of motion for the complex coordinates of the electronic order parameter (EOP, $\tilde{{\rm{\Delta }}}$) with linear coupling to the bare phonons (${\tilde{\xi }}_{n}$, Fig. 1). This provides a versatile framework to include effects such as time-dependent perturbations of the potentials and higher spatial dimensions^12, 13^, which should find more general applicability to other collective phenomena and photo-induced phase transitions.

In such optical-pump optical-probe (OP-OP) experiments^11–13^ one specifically probes the amplitude channel via coherent oscillations in the interband permittivity. While the phase channel has been probed in certain CDW materials with terahertz (THz) and infrared spectroscopy^10^ including non-equilibrium dynamics^14, 15^, these studies did not interpret the results in terms of the QM or TDGL models. However, for further development and testing of the theoretical description, the phase channel plays a crucial role: involving the translation of the CDW condensate, it is more directly related to the non-linear transport^16^ and should be more sensitive to effects such as impurities, intra- and inter-chain interactions^17–20^. Moreover, a close inspection of the nominal QM^8, 9^ and TDGL^11, 12^ models reveals *qualitative* differences in the predictions specifically for the phase modes, even for *T* ≈ *T*_c_ (as addressed below). Hence, further experimental studies of the phase-channel and theory development are required to reach a complete and unified description of the CDW physics.

Towards this goal, in the present paper we target the quasi-1D conductor blue bronze (K_0.3_MoO_3_)^21^, which forms an incommensurate CDW below *T*_c_ = 183 K, and whose low-energy spectrum has been intensely studied via neutron scattering^22, 23^, Raman^24, 25^, and far-infrared^10, 26^ spectroscopy. Moreover, the non-equilibrium response following femtosecond optical excitation has been studied recently using optical^12, 13, 27–29^, photoelectron^30^ and electron-diffraction^31^ probes, including application of the TDGL model to the amplitude modes^11–13^. For the phase modes, while previous GS (far-)IR measurements covered a wide frequency range^10, 26^, these were confined to low temperature (≤10 K). Moreover, to our knowledge, no studies of the non-equilibrium response of the phase-phonons have been reported (although a signature assigned to the phason below 50 GHz was resolved in OP-OP measurements^32^). Here we apply optical-pump THz-probe spectroscopy (OP-TP), which probes the phase-phonons via the broadband, transient *complex* conductivity, and reconcile both the non-equilibrium dynamics and *T*-dependence of the bands with a generalized TDGL model.

## Results

### Non-equilibrium dynamics

**Figure 2:**
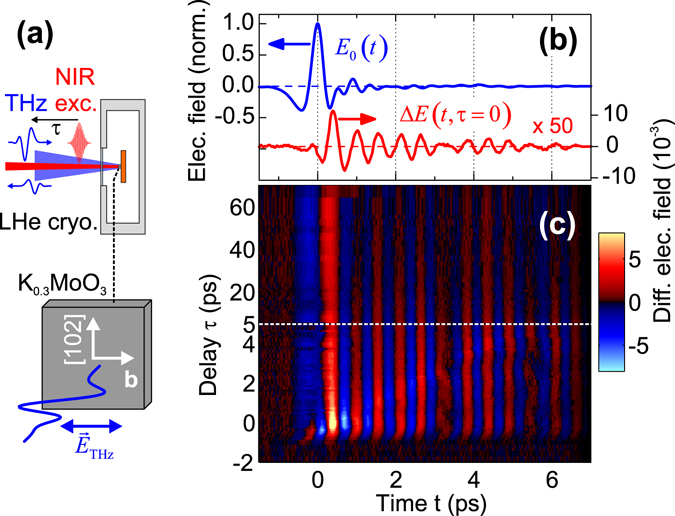
Time-domain field response to optical excitation. (**a**) Experimental OP-TP reflection geometry. (**b**) Detected temporal electric fields (*T* = 50 K, pump fluence *F*_ex_ = 550 *μ*J cm^−2^): Reference field *E*_0_(*t*) (reflected signal without optical excitation); differential field change Δ*E*(*t*, *τ*) for pump-probe delay *τ* = 0. (**c**) Full 2D map of Δ*E*(*t*, *τ*). Note that the data is plotted with two different scales for the ranges *τ* ∈ [−2, 5] ps and *τ* ∈ [5, 70] ps, respectively.

**Figure 3:**
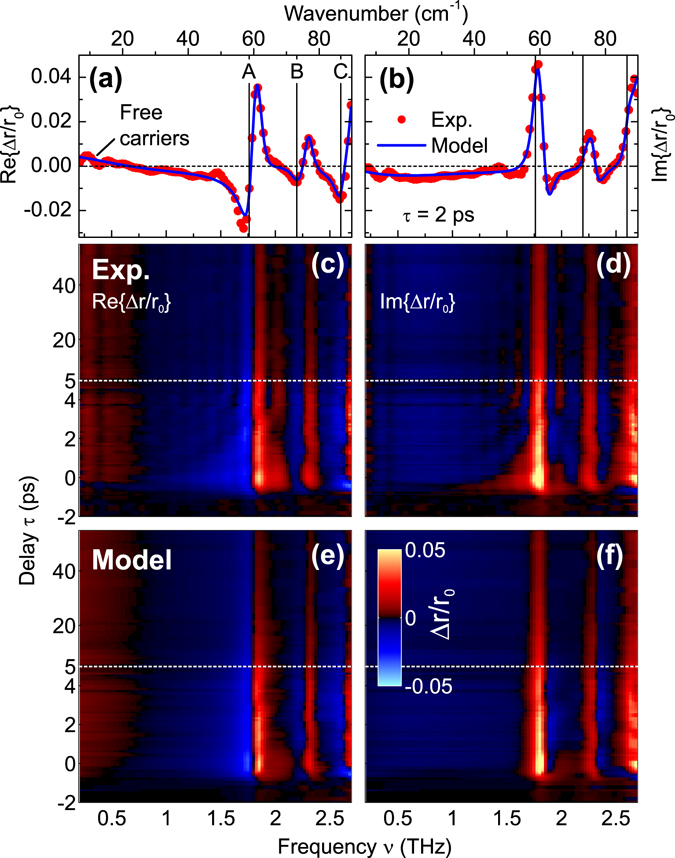
Time-resolved phase-phonon spectra and model analysis. Differential field reflectivity spectra Δ*r*/*r*_0_ ∝ Δ*σ*/(*ε*_r_ − 1) at *T* = 50 K (left graphs Re{Δ*r*/*r*_0_}; right graphs Im{Δ*r*/*r*_0_}). (**a**,**b**) For pump-probe delay *τ* = 2 ps, including both experimental data (points) and fitted model (see text). The GS frequencies of the three main bands (labeled A–C) from the OP-TP analysis are included as vertical lines. (**c**,**d**) Full experimental data Δ*r*(*ν*, *τ*)/*r*_0_(*ν*) obtained from Fourier analysis of each row in Fig. 2(c); (**e**,**f**) corresponding model fits.

The OP-TP experiments were performed with both optical-pump (*hν*_ex_ = 1.6 eV) and THz-probe pulses (time delay *τ*) normally incident on the sample (Fig. 2(a)), and the reflected THz field was measured with time-domain sampling (see Methods section) In Fig. 2(b) we show examples of the reflected reference pulse *E*_0_(*t*) and photoinduced differential signal Δ*E*(*t*, *τ*) = *E*(*t*, *τ*) − *E*_0_(*t*) at *τ* = 0 (with the THz field polarized parallel to the crystal b-axis) for *T* = 50 K and an excitation fluence of *F*_ex_ = 550 *μ*J cm^−2^. The full 2D signal built up from a sequence of measurements vs. *τ* is shown in Fig. 2(c). One sees that the temporal onset of Δ*E* is shifted somewhat from the main peak of *E*_0_ and exhibits a ringing signature composed of multiple beating frequencies due to a perturbation of the GS phase-phonons. The corresponding differential reflectivity spectra Δ*r*(*ν*, *τ*)/*r*_0_(*ν*) are presented in Fig. 3(a–d). Accounting for the short excitation depth *D*_ex_ = 1/*α*_ex_ compared to the THz probe wavelengths, one can show that the complex conductivity change Δ*σ*(*ν*, *τ*) is essentially in-phase with Δ*r*/*r*_0_ (see Supplemental Information), and hence spectral features in Δ*r*/*r*_0_ correlate closely with those in Δ*σ* (with minor distortions due to a pre-factor containing the GS permittivity). The spectra in Fig. 3(a) contain a sequence of derivative-shape features in the range $\mathop{ > }\limits_{ \tilde {}}$1.5 THz for Re{Δ*r*/*r*_0_} (*τ* = 2 ps), whose polarity implies a photoinduced *blue*-shift of existing bands. The most dominant feature (with a zero-crossing at 1.78 THz) occurs about a frequency where a weak spectral modulation was indeed observed previously^10^ but not assigned as a phase-phonon. From additional GS THz measurements and the analysis below, we deduce that the signatures arise from three main bands (labeled A–C from hereon, as shown in Fig. 3(a)), where bands (B, C) correspond to those previously found in FTIR measurements^10^. The weak signature at low frequency can be fit well with a Drude model (see below), which we assign to the generation of mobile carriers. From the full 2D data (Fig. 3(c,d)) one sees that all features persist for delays beyond 50 ps, after initial relaxation in the first few ps.

**Figure 4:**
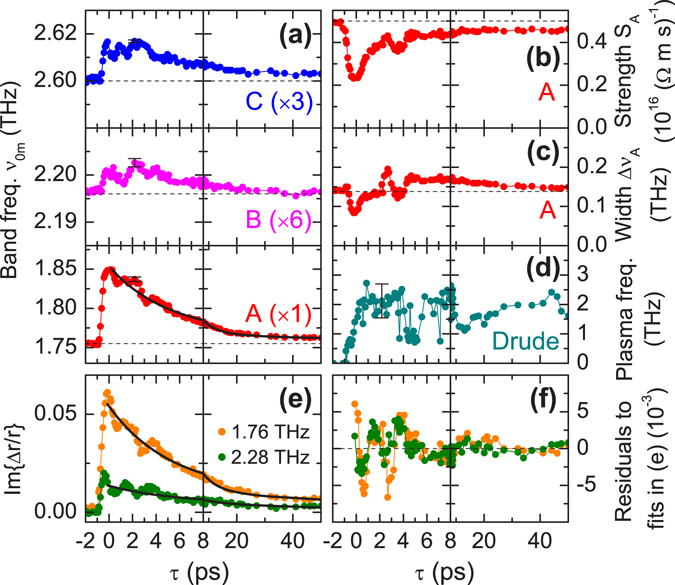
Parameter kinetics for phase-phonon bands and free-carrier response. Results of fitting Δ*r*/*r*_0_ data in Fig. 3 (*T* = 50 K) vs. pump-probe delay *τ*. (**a**) Band frequencies *ν*_0*n*_ for the three bands *n* = A,B,C. GS values obtained during global fit indicated by dashed horizontal lines. (**b**,**c**) Band strength and bandwidth (FWHM) for Band A, respectively. (**d**) Fitted Drude plasma frequency *ν*_pl_ for free-carrier contribution. Note that the fluctuations at later delays arise due to small drifts and noise during the measurement run, and any structure should rather be attributed to the non-monotonic order of delays used for the measurement run. (**e**) Experimental kinetics of Im{Δ*r*/*r*_0_} at two selected frequencies, as indicated, and global bi-exponential fit (solid curves). (**f**) Corresponding residual from fitting in (**e**). Representative confidence intervals in the fitted frequency shifts and plasma frequency indicated by the error bars in (**a**,**d**), respectively (see Fig. 5(b,d) for errors in the GS parameters).

In order to disentangle the band contributions and extract parameter kinetics vs. *τ*, we fit the full complex data based on photoinduced modification of three Lorentzian bands and a Drude contribution (see Methods section). The resulting fitted spectra are shown in Fig. 3(e,f) (and also as curves in Fig. 3(a,b) for *τ* = 2 ps), which reproduce the experimental data well, except for the additional broadening seen in the first 2 ps. Simulations for a single perturbed phonon show that this transient broadening can be attributed to the response of a time-non-stationary medium^33^ due to sub-ps dynamics (see Supplemental Information), and hence cannot be reproduced using the quasi-stationary spectral analysis here. In Fig. 4(a–d) we plot selected fitted band parameters as a function of delay *τ*, where the blue-shifts for all bands A–C are clearly evident (*ν*_0*n*_(*τ*), Fig. 4(a)). For band A, this is accompanied with a transient reduction in band strength (*S*_A_, Fig. 4(b)), and additional broadening (after initial development in the first few ps, Fig. 4(c)). As expected from inspection of the low-frequency region in Fig. 3(c), the fitted Drude plasma frequency rises to a fairly constant plateau at *ν*_pl_ = 2 THz (Fig. 4(d)) with a scattering time *τ*_s_ ~ 90 fs. Based on the low-frequency value of ${\varepsilon }_{{\rm{r}}}(\nu \to 0) \sim 100$, *ν*_pl_ corresponds to an excitation density ${N}_{{\rm{ex}}}/({m}_{{\rm{eff}}}/{m}_{0}) \sim 5\times {10}^{18}\,{{\rm{cm}}}^{-3}$. As these photoexcited carriers may well populate orbitals distinct from those of the CDW condensate, we do not necessarily associate the effective mass here with that of the CDW (${m}_{{\rm{eff}}}^{{\rm{CDW}}} \sim 300\cdot {m}_{0}$ ^21^).

To extract kinetic time scales, we fit the experimental data directly, i.e. the kinetics of Im{Δ*r*/*r*_0_} for *ν* = 1.76 and 2.28 THz (the two peaks in Fig. 3(b)) as shown in Fig. 4(e). A global bi-exponential fit yielded time constants of *τ*_1_ = 5.1 ps and *τ*_2_ = 60 ps, in addition to a small but significant, long-lived offset. The values *τ*_1,2_ also reproduce the band-shift kinetics well (solid curve in Fig. 4(a) for band A). The fit residuals (Fig. 4(f)) exhibit roughly two oscillation cycles with period ~2 s followed by a weaker oscillation with period ~10 ps. This additional slow modulation of the system, which is in-phase for both bands, presumably results from coherent charge/lattice oscillations polarized perpendicular to the surface. As discussed below, we attribute the blue-shifts to a reduction of the e-ph coupling due to the presence of the free carriers (i.e. a reduction of the red-shift of the dressed phonons). As the plasma frequency *ν*_pl_(*τ*) shows no such decay kinetics, this would imply that after the initial reaction of the phonons to the free charges, the system can reconfigure in such a way that the e-ph interactions can be partially reestablished. This process may again involve relaxation of excitation distributions perpendicular to the surface. Compared to OP-OP studies^11, 28^ of the amplitude-phonons (and interband electronic contributions), our value of *τ*_1_ is consistent with a component in the range 5–10 ps^11, 12, 29^ which was assigned to a second stage of CDW recovery, whereas the initial sub-ps CDW recovery (on the same time scale as the period of the THz waves here) would rather contribute to the transient broadening, as seen in our data. A persistent offset in the (electronic) OP-OP signals was attributed tentatively to residual cooling^28^, although our results here indicate that a long-lived free-carrier population may well contribute.

### Temperature dependence and extended Ginzburg-Landau model

**Figure 5:**
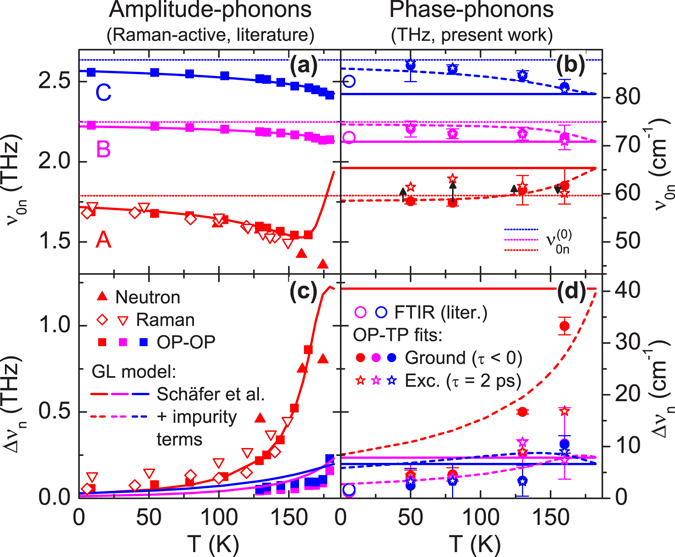
Temperature dependence of amplitude- and phase-phonon band parameters. Amplitude- (left graphs) and phase-phonons (right) for *T* < *T*_c_; center frequencies *ν*_0*n*_ (top graphs) and bandwidths Δ*ν*_*n*_ (FWHM, bottom), for bands *n* = A (red), B (magenta) and C (blue). Literature data from neutron scattering ($\blacktriangle$ ^23^), Raman scattering ($\{\backslash triangledown\}$ ^24^; $\diamond$ ^25^), optical-pump optical-probe reflectivity ($\blacksquare$ ^11, 12^), and far-infrared FTIR measurements (○^10^, *T* = 6 K). THz TDS data (present work) from global analysis of OP-TP data (for *T* = 50, 80, 130 and 160 K) for both GS (●) and *τ* = 2 ps after excitation (☆), including confidence intervals for the GS parameters. Also included are model curves from the TDGL model, with parameters fit to the amplitude phonons in ref. 11, both without (solid) and with (dashed) pinning/scattering contributions from impurities (with Ω_i_(0)/2*π* = 9.3 THz and *g*_i_ = 1.9, see text; note that this only affects the predictions for the phase channel, so both curves are superposed for the amplitude channel).

We turn now to the temperature dependence of the phase-phonons, obtained from measurements as per those above for *T* = 50, 80, 130 and 160 K (see Supplemental Information for full data sets), which yielded the fitted band frequencies *ν*_0*n*_ and widths Δ*ν*_*n*_ shown in Fig. 5 (right) for both the GS, and excited state for *τ* = 2 ps. One sees a clear softening for band B and C, but rather a stiffening for band A, with increasing *T*, accompanied with significant broadening for band A. The GS data for bands B, C from ref. 10 at *T* = 6 K are also included, and are reasonably consistent with an extrapolation of our parameters (recall that band A was not assigned in ref. 10).

Literature data for the amplitude modes are also shown in Fig. 5 (left), comprising GS neutron- and Raman-spectroscopy for band A, and OP-OP data for all three bands (sources cited in caption), which are reasonably consistent (except for the deviation between neutron and OP-OP data for *ν*_0A_ approaching *T*_c_). Also included are the theoretical curves based on the TDGL model adopted from refs 11, 12, which demonstrates the very good agreement obtained by those authors (even for $T\ll {T}_{{\rm{c}}}$). However, the nominal TDGL model predicts *T*-*independent* phase-phonon bands (which coincide with the amplitude modes for *T* → *T*_c_; solid horizontal lines in Fig. 5(b,d)), which clearly is not consistent with our results away from *T*_c_. While any GL treatment^2^ is only a first-order expansion about *T* ≈ *T*_c_, it is not clear why it should break down here specifically for the phase channel. Hence we considered possible *T*-dependent effects which should only affect the phase channel (Δ_2_, *ξ*_*n*2_) in the TDGL equations. A straightforward generalization which reproduces the observed trends for all bands is based on impurity effects (see Supplemental Information), which dominantly affect the CDW *phase* channel via pinning^17, 34, 35^ and scattering^18, 36^. We incorporated these via a pinning potential ${U}_{{\rm{i}}}=-\,\tfrac{1}{2}{{\rm{\Omega }}}_{{\rm{i}}}^{2}(T){{\rm{\Delta }}}_{2}^{2}$ and damping term *γ*_2_ → *γ* + *γ*_i_(*T*), taken to depend on the relative equilibrium EOP amplitude *δ*_0_(*T*) = (1 − *T*/*T*_c_)^1/2^ as per ${{\rm{\Omega }}}_{{\rm{i}}}^{2}(T)={{\rm{\Omega }}}_{{\rm{i}}}^{2}(0){\delta }_{0}^{n}(T)$ and ${\gamma }_{i}(T)/\gamma ={g}_{{\rm{i}}}{\delta }_{0}^{n}(T)$, where the exponent *n* = 2 was required to obtain reasonable agreement with the data. As can be seen in Fig. 5(b,d), the generalized model reproduces the qualitative trends in the phase-phonon frequencies *ν*_0*n*_(*T*) (including the crossing of *ν*_0A_ through ${\nu }_{0{\rm{A}}}^{(0)}$ at *T* ~ 120 K) and significantly improves the predicted trend for Δ*ν*_A_(*T*), while maintaining all other TDGL parameters used for the amplitude-phonons. The exponent *n* = 2 deviates from the value *n* = 1 which is commonly taken for the potential of a single impurity: ${U}_{{\rm{i}}}\propto {{\rm{\Omega }}}_{{\rm{i}}}^{2}\propto {\delta }_{0}$ ^17, 34, 35^. However, QM treatments have predicted impurity pinning forces which grow faster than Δ_0_(*T*)^37, 38^ due to competition between the CDW and formation of Friedel oscillations about each impurity. Also, a divergence in phase damping with decreasing *T* was deduced from non-linear transport experiments^16^ and was discussed in terms of screening^39^ and impurity-induced phase deformations of the CDW, which enhance phase scattering as compensating thermal carriers are frozen out (which dominates over the damping due to the corresponding CDW-carrier scattering^19^ which would instead grow with *T*).

Considering the photoinduced band changes, one can see that at least qualitatively, the observed blue-shifts are consistent with a reduction of the couplings *m*_*n*_ between EOP and bare phonons^11^ (shifting the frequencies *ν*_0*n*_ in the direction of ${\nu }_{0n}^{(0)}$), which we attribute to the presence of the long-lived free carriers. The fact that *ν*_0A_ shifts even above ${\nu }_{0{\rm{A}}}^{(0)}$ for *T* < 160 K (Fig. 5(b)) may be due to an additional partial suppression of the impurity pinning (recall that the solid lines are the predicted frequencies in the absence of pinning). This raises the question whether the amplitude-phonons in OP-OP measurements are also blue-shifted^11–13^. A study vs. fluence *F*_ex_^29^, however, revealed a small *red*-shift at high fluence. One mechanism which could account for this different behavior involves relaxing the strict phase coherence of the coupling between EOP and bare phonons after excitation,

$${U}_{n}\to -\,{m}_{n}{\rm{\Delta }}\cdot {\xi }_{n}[(1-\eta )\,\cos \,(\phi -\chi )+\eta ],\,(0\le \eta \le 1)$$

e.g. due to the presence of inhomogeneous distortions of the CDW. This modification only reduces the effective coupling for the phase channel (see Supplemental Information), consistent with the experimental findings.

## Discussion

While the extended TDGL model allows us to assign and account for all three phase-phonons here, it is important to note that this contrasts with the predictions of the nominal QM theory (even for *T* ≈ *T*_c_)^8, 9^, where one of the renormalized modes is driven to a near-zero-frequency “phason” (which was experimentally assigned^10^ at *ν* ~ 0.1 THz for K_0.3_MoO_3_). This discrepancy manifests much more clearly for the phase channel, as all amplitude-phonons are predicted to remain at finite frequencies in both models. A key difference appears to lie in the treatment of electronic damping in the QM model (see Supplemental Information), which in the phenomenological TDGL treatment is dictated by free parameters chosen to agree with experiment (corresponding to an overdamped EOP^11, 12^). This motivates further theory development to include additional interactions in deriving the spectral response function. A first step was taken in this direction with extensions of the QM model to include long-range Coulomb effects^19, 20^, which were shown to strongly affect the phase channel, causing spectral weight to be transferred from the phason to an additional finite-frequency resonance.

This study demonstrates how coherent THz spectroscopy can provide unique insight into the underlying CDW physics, such as impurity and screening effects which predominantly affect the phase channel. The inclusion of these effects within a generalized TDGL model can account for the *T*-dependence and photoinduced dynamics observed here for K_0.3_MoO_3_. Given that the TDGL model provides a highly practical framework for interpretation of (non-equilibrium) experiments^11–13^, we strongly advocate further investigations into its applicability and correspondence to microscopic QM treatments.

## Methods

The single crystals of K_0.3_MoO_3_ were grown according to the temperature-gradient flux method^40^, reaching facial dimensions up to 4 × 5 mm^2^, and the crystal structure and orientation were confirmed by x-ray diffraction on a small single crystal. The crystal surfaces were sufficiently flat/smooth to be used in the experiments as-grown.

The terahertz time-domain spectroscopy system used for the optical-pump THz-probe experiments is shown in the Supplemental Information. The system is based on a 1-kHz Ti:Al_2_O_3_ amplifier laser (Clark-MXR CPA-2101, *λ*_ex_ = 775 nm, pulse duration ~150 fs FWHM). The THz pulses were generated by a large-area InAs surface emitter, while a 0.5-mm-thick 〈110〉-cut ZnTe is used for electrooptic detection (frequency range ~0.2–3 THz). The blue bronze samples were mounted in a liquid-helium cryostat (Oxford Microstat) and could be oriented with the conductive b-axis either parallel or perpendicular to the p-polarized THz probe field. A thin polypropylene film (50-*μ*m-thick, Goodfellow PP301350) was used as the window to minimize any distortion of the THz beam (and two-photon absorption effects for the optical excitation pulse). In order to realize a normal-incidence reflection geometry, a two-way beamsplitter (0.5-mm-thick high-resistivity Si wafer) is placed in the THz beam before the sample. The optical excitation beam for the sample passes through a small hole in the sample focusing mirror (off-axis paraboloidal mirror, effective focal distance *f*_eff_ = 101.6 mm), brought to a weak focus on the sample (with a spot diameter of ~2 mm) using a telescope. While measurements were performed with the THz field polarized both parallel and perpendicular to the *b*-axis, the phase-phonon signals presented here were observed only in the former case. Also, measurements were made with fluence in the range *F*_ex_ = 140–720 *μ*J cm^−2^, although we present here only data for *F*_ex_ = 550 *μ*J cm^−2^ which yielded a favorable compromise between signal strength and saturation effects^29^. The mechanical delay stage for THz detection was placed in the emitter arm, such that the time-variable *t* corresponds to a fixed pump-probe delay *τ* for each point in the acquired THz waveform^41^. Besides yielding a 2D pump-probe signal Δ*E*(*t*, *τ*) = *E*(*t*, *τ*) − *E*_0_(*t*) with a synchronous zero-delay for all *t*, this also rejects any pump-only emission signals in the resulting spectral data Δ*E*(*ν*, *τ*) (which were also observed). A dual-chopping scheme with two lock-in amplifiers was employed to synchronously measure Δ*E* and *E*_0_^42^, which was essential to track phase drifts in the reflected THz field due to unavoidable longitudinal movement of the cryostat (of some 10 μm) during measurements.

The fit model for each complex field reflectivity spectrum Δ*r*(*ν*, *τ*)/*r*_0_(*ν*) in the OP-TP data was based on assuming three Lorentzian bands and a Drude contribution (as well as a frequency-independent background permittivity *ε*_br_ to account for higher-lying resonances), whose GS parameters (frequency *ν*_0*n*_, width Δ*ν*_*n*_ and strength *S*_*n*_) are modified by the optical excitation to ${\nu }_{0n}^{^{\prime} }(\tau )$, ${\rm{\Delta }}{\nu }_{n}^{^{\prime} }(\tau )$, ${S}_{n}^{^{\prime} }(\tau )$ and ${\varepsilon }_{{\rm{br}}}^{^{\prime} }(\tau )$. By definition, *ν*_0,D_ ≡ ${\nu }_{0,{\rm{D}}}^{^{\prime} }$ ≡ 0 for the Drude contribution, and we assumed no free carriers in the GS (*S*_D_ = 0). Note that we also tested the use of Fano lineshapes (as was applied in the vicinity of a single band in an OP-TP study on 1*T*-TaS_2_^14^), especially as this could be appropriate for the proposed interaction with free carriers. However, the Fano interaction (which essentially only mixes the real and imaginary parts of the band conductivity) could not reproduce such large shifts, especially for band A. Due to baseline/resolution issues in the GS THz-TDS results, we included global GS band parameters in the fit procedure (the fitted GS band strengths were constrained to remain comparable to those from GS THz-TDS data). In order to implement a robust and practical fitting scheme including the GS parameters, we first selected spectra from a small number of delays (*τ*_*n*_ = 2, 10 ps), and performed the fitting of all parameters. Thereafter, the GS parameters were held fixed and each spectrum was fitted sequentially for the excited-state parameters. At each iteration, we first used an approximate expression for Δ*r*/*r*_0_ valid for ${D}_{{\rm{ex}}}\ll {\lambda }_{{\rm{THz}}}$ (Eq. 1 in Supplemental Information) to estimate the model differential field reflectivity. While this equation is nonlinear in the GS complex permittivity, it is linear in the differential change. This allowed to determine the ES band strengths ${S}_{n}^{^{\prime} }(\tau )$ via generalized regression, which relieved the fitting algorithm of a fraction of the search parameters and facilitated the fitting procedure. Then the exact expression for Δ*r*/*r*_0_ was calculated and used to compute the current misfit. To avoid stagnation, the nonlinear optimization was based on Covariance Matrix Adaptation Evolution Strategy (CMA-ES), followed by local refinement with the Nelder-Mead simplex method. Confidence intervals in the fitted parameters were obtained from the numerical Hessian of the misfit function, corresponding to ±1*σ*. Note that the excitation depth *D*_ex_ was also fitted (as the nominal value from linear measurements could not account for the experimental data, see main paper). However, after the optimum value was determined with the data for *T* = 50 K, it was kept fixed for all other temperatures *T* = 80, 130, 160 K.

### Data availability

The raw data and analysis results of the current study are available from the corresponding author on reasonable request.

## Electronic supplementary material

Supplemental Information
