# Supplementary material for: Phase-channel dynamics reveal the role of impurities and screening in a quasi-one-dimensional charge-density wave system
Source: Sci Rep. 2017 May 17;7:2039. doi: 10.1038/s41598-017-02198-x (PMC5435704; doi:10.1038/s41598-017-02198-x)
Supplement: Supplementary file 1 — Supplemental Information [file 41598_2017_2198_MOESM1_ESM.pdf]

Supplemental Information to: "Phase-channel dynamics reveal the role of impurities and screening in a quasi-one-dimensional charge-density wave system"

M. D. Thomson,[1] K. Rabia,[1] F. Meng,[1] M. Bykov,[2] S. van Smaalen,[2] and H. G. Roskos[1]

[1]*Physikalisches Institut, J. W. Goethe-Universität, 60438 Frankfurt am Main, Germany*
[2]*Laboratory of Crystallography, University of Bayreuth, 95440 Bayreuth, Germany*

A. Experimental setup

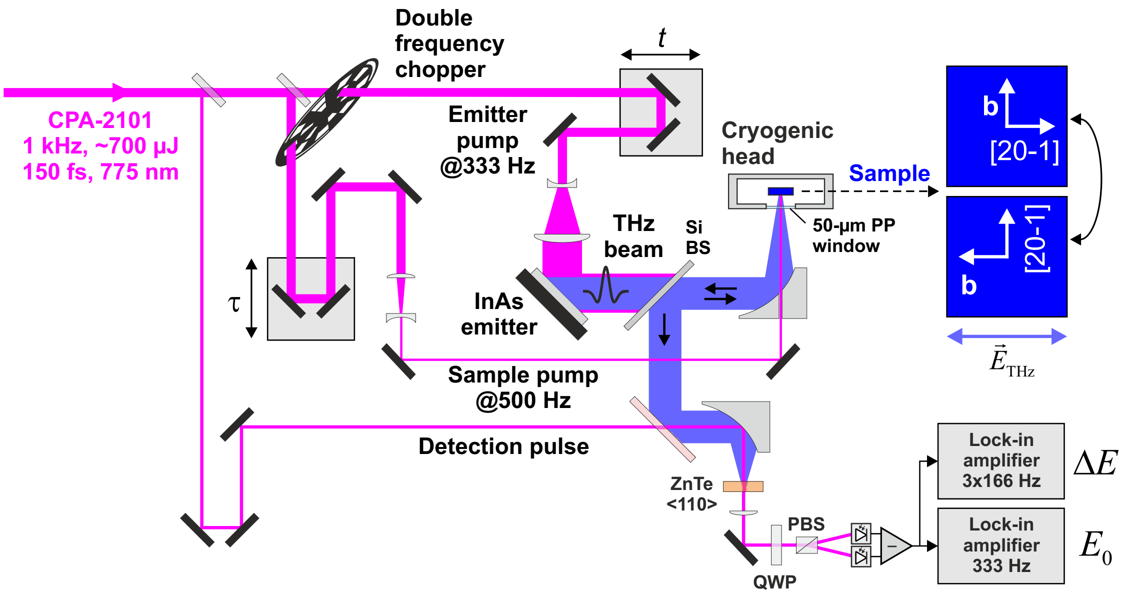

FIG. 1. Schematic of full experimental setup for optical-pump THz-probe measurements, as described in the main paper.

B. Derivation of approximate relation between differential reflectivity and conductivity for an exponential excitation profile

The complex field reflection coefficient for a sample where the excitation depth is small compared to the probe wavelength (as is the case for blue bronze with near-infrared excitation and THz probing) deviates significantly from the Fresnel formula for a homogeneous medium [1, 2]. For a pump-induced change in the sample complex permittivity $\Delta\varepsilon_r(\omega,z) = \Delta\varepsilon_r(\omega)e^{-z/D_{ex}}$ (where $D_{ex} = 1/\alpha_{ex}$ is the excitation depth) the reflectivity can still be expressed in the form $r = (1-n')/(1+n')$ where $n' = n + \sqrt{\Delta\varepsilon_r}X_\beta(\xi)$, $n = \sqrt{\varepsilon_r}$ is the background (unpumped) refractive index, and $X_\beta(\xi) = I_{\beta+1}(\xi)/I_\beta(\xi)$ is the ratio of modified Bessel functions with $\beta = 2i\omega n D_{ex}/c$ and $\xi = 2i\omega\sqrt{\Delta\varepsilon_r}D_{ex}/c$. For $D_{ex} \ll \lambda_{THz}$, one can approximate the expression for $X_\beta(\xi)$ to first-order as: $X_\beta(\xi) \to \frac{1}{2}\xi/(\beta+1)$. Substituting this into the expression for r, noting that the ground-state reflectivity $r_0 = (1-n)/(1+n)$, one can readily derive (assuming $\Delta n = n' - n \ll n$):

$$\frac{\Delta r}{r_0} \approx \frac{2ik_0D_{ex}}{1+2ik_0D_{ex}\sqrt{\varepsilon_r}}\frac{1}{(\varepsilon_r-1)}\Delta\varepsilon_r \approx \frac{2D_{ex}}{1+2ik_0D_{ex}\sqrt{\varepsilon_r}}\frac{1}{\varepsilon_0 c(\varepsilon_r-1)}\Delta\sigma, \quad (1)$$

where $\Delta r = r - r_0$, $k_0 = \omega/c$, and $\Delta\sigma = i\varepsilon_0\omega\Delta\varepsilon_r$ is the pump-induced change in complex conductivity. The validity of these approximations was thoroughly checked in numerical tests with parameters appropriate to the experiments presented in the main paper. Note that if one were instead to use a simple Fresnel reflection treatment assuming homogeneous excitation vs. depth, this introduces an imaginary unit in the coefficient relating $\Delta r/r_0$ and $\Delta\sigma$, such that real and imaginary parts would be erroneously exchanged in calculating $\Delta\sigma$ from the data.

C. Complete set of transient spectra and fits for all temperatures

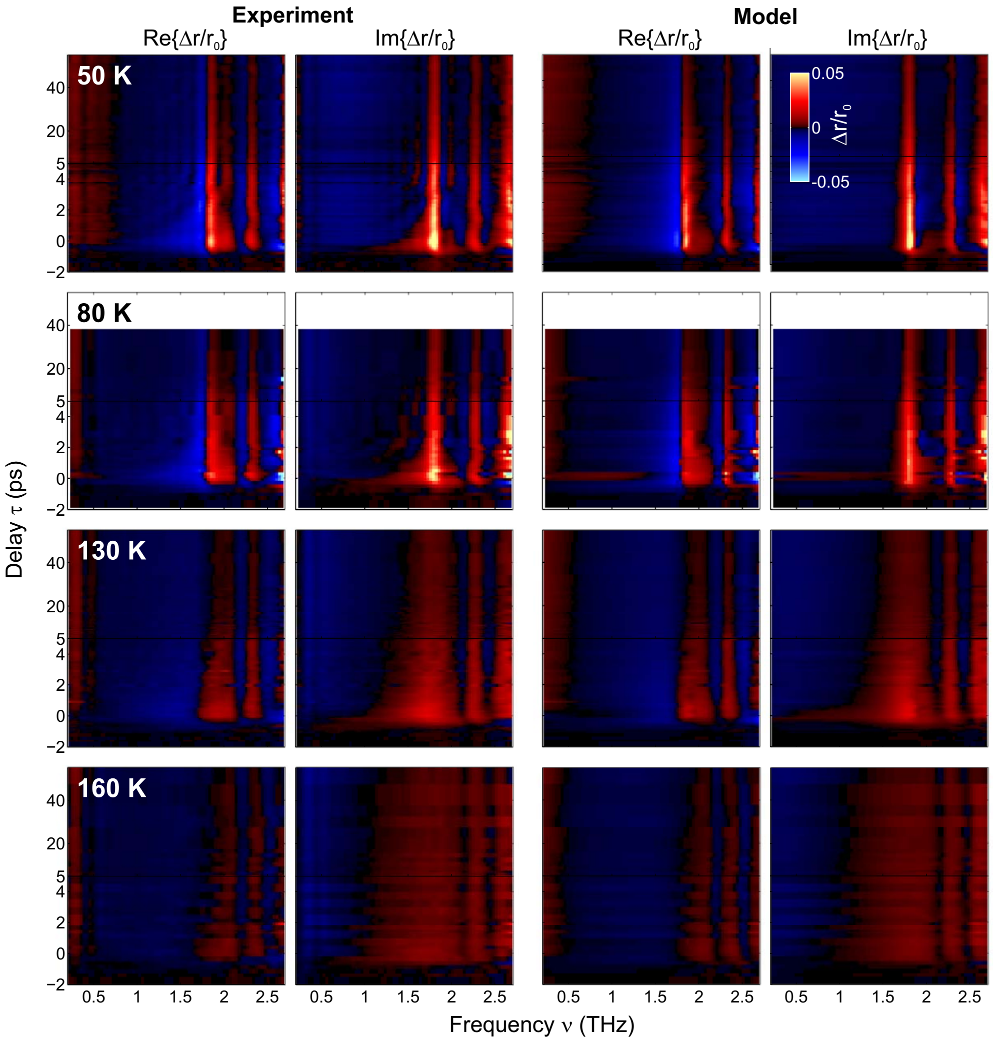

FIG. 2. Transient reflectivity spectra for all measured temperatures $T = 50, 80, 130, 160$ K with excitation fluence $F_{ex} = 550\,\mu\mathrm{J\,cm^{-2}}$ (as per data in main paper for $T = 50$ K (Fig. 3)). Experimental data (left two columns) and Lorentzian-Drude fits (right two columns); real and imaginary parts of $\Delta r/r_0$ as indicated.

D. Application of the time-dependent Ginzburg-Landau treatment

Here we consider generalizations of the time-dependent Ginzburg-Landau (TDGL) model prescribed in [3, 4]. For completeness, we first briefly summarize this, in order to clarify the notation and modifications

that follow. We consider the potential function, in terms of the complex electronic order parameter $\tilde{\Delta} = \Delta e^{i\varphi} = \Delta_1 + i\Delta_2$ and complex bare phonon coordinates $\tilde{\xi}_n = \xi_n e^{i\chi_n} = \xi_{n1} + i\xi_{n2}$ ($n = 1 \ldots N$) (where all coordinates refer to the complex envelope amplitudes of the $q = 2k_F$ components) as per:

$$U(\tilde{\Delta}, \tilde{\xi}_1, \ldots, \tilde{\xi}_N) = U_\Delta + U_{\xi n} + U_c \tag{2}$$

where $U_\Delta = -\frac{1}{2}\alpha(T_{c0} - T)\Delta^2 + \frac{1}{4}\beta\Delta^4$ is the Mexican hat potential, $U_{\xi n} = \frac{1}{2}\omega_{0n}^2\xi_n^2$ represents the elastic energy stored in the bare phonon mode n with frequency ω_{0n}, and $U_c = -m_n(\Delta_1\xi_{n1} + \Delta_2\xi_{n2}) = -m_n\Delta\cdot\xi_n\cos(\varphi - \chi_n)$ is the linear coupling term (summations over n are left implicit). This has the equilibrium solution $\Delta_0^2 = \frac{\alpha(T_c - T)}{\beta}$ and $\xi_{0n} = \frac{m_n}{\omega_{0n}^2}\Delta_0$, where $T_c = T_{c0} + \frac{m_n^2}{\alpha\omega_{0n}^2}$ is the renormalized critical temperature.

Taking $\varphi_0 = \chi_{n0} = 0$ as the equilibrium phase (at first, arbitrarily, as there is no pinning term and hence U depends only on $(\varphi - \chi_n)$), and calculating the Hessian matrix of the potential about $\tilde{\Delta}_0 = \Delta_0$ yields the linearized equations of motion [3, 4]:

$$\partial_t^2\hat{\Delta}_1 = -\left[2\alpha(T_c - T) + \frac{m_n^2}{\omega_{0n}^2}\right]\hat{\Delta}_1 + m_n\hat{\xi}_{n1} - \gamma_1\partial_t\hat{\Delta}_1 \tag{3a}$$

$$\partial_t^2\hat{\Delta}_2 = -\frac{m_n^2}{\omega_{0n}^2}\hat{\Delta}_2 + m_n\hat{\xi}_{n2} - \gamma_2\partial_t\hat{\Delta}_2 \tag{3b}$$

$$\partial_t^2\hat{\xi}_{n1} = m_n\hat{\Delta}_1 - \omega_{0n}^2\hat{\xi}_{n1} \tag{3c}$$

$$\partial_t^2\hat{\xi}_{n2} = m_n\hat{\Delta}_2 - \omega_{0n}^2\hat{\xi}_{n2} \tag{3d}$$

where $\hat{\Delta}_1 \approx \Delta - \Delta_0$ and $\hat{\Delta}_2 \approx \Delta_0\varphi$ represent the amplitude and phase deviations from equilibrium (likewise for $\hat{\xi}_{n1}$,$\hat{\xi}_{n2}$), and we have added phenomenological damping constants $\gamma_{1,2}$ for $\hat{\Delta}_{1,2}$. Note that while in a classical treatment one has $\gamma_1 = \gamma_2 = \gamma$ [5], we allow here for $\gamma_1 \neq \gamma_2$ to account for different quasi-particle scattering channels. Moreover, the variables are normalized to unit mass and we do not include any inherent damping for the bare phonon modes (which did not assist in fitting the phase-phonon data). The equations for the amplitude (Raman-active) and phase (IR-active) channels are decoupled, and converge to the same set of solutions for $T \to T_c$ (corresponding to the T-independent phase channel).

In the overdamped limit for the EOP (as adopted in [3, 4]), i.e. $\partial_t^2\Delta_j \ll \gamma\partial_t\Delta_j$ (which is equivalent to neglecting the inertial mass), Eq.s 3(a-b) become:

$$\partial_t\hat{\Delta}_1 = -\kappa_1\left[2\alpha(T_c - T) + \frac{m_n^2}{\omega_{0n}^2}\right]\hat{\Delta}_1 + \kappa_1 m_n\hat{\xi}_{n1} \tag{4a}$$

$$\partial_t\hat{\Delta}_2 = -\kappa_2\frac{m_n^2}{\omega_{0n}^2}\hat{\Delta}_2 + \kappa_2 m_n\hat{\xi}_{n2} \tag{4b}$$

where $\kappa_j = \gamma_j^{-1}$ is the scattering time. As per [3, 4], one can solve these linear equations with the ansatz $\propto e^{\lambda t}$ for the eigenvalues $\lambda_k = -\Gamma_k/2 + i\Omega_{0k}$ (and corresponding eigenvectors, which reflect the relative contribution of the EOP and each phonon to the mode), which yields either $N + 2$ (general damping, Eq. 3) or $N + 1$ (overdamped limit, Eq. 4) modes for each channel (after discounting for the complex-conjugate solutions with $\Omega_{0k} < 0$). For each channel, one obtains a set of N finite-frequency modes which, at least for the parameters used for $K_{0.3}MoO_3$ in [3, 4], each contain a dominant contribution from one of the bare modes, and hence have frequencies Ω_{0k} only moderately shifted from a given bare phonon at ω_{0n} (although in general, stronger mixing and avoided crossings can result [4]). In the overdamped limit, the remaining eigenvalues have $\Im(\lambda) = 0$, and also $\Re(\lambda) = 0$ for the phase channel (and amplitude channel for $T \to T_c$). The latter was associated in [3, 4] with a true soft mode of the system [6]. As the authors asserted, this indeed represents a distinct physical picture to that from earlier quantum-mechanical (QM) treatments, as discussed below.

			n	
		1	2	3
$\omega_{0n}/2\pi$	(THz)	1.79	2.25	2.64
κm_n^2	(10^{36} s^{-3})	580	320	1150
$\gamma_1^{-1} = \gamma_2^{-1}$	(fs)		3.4	
α	(ps^{-2} K^{-1})		46	

TABLE I. Fitted parameters TDGL parameters used both to reproduce the Raman channel data from [3] and as the basis for the IR-active data measured in the present paper.

In order to provide a starting point for the analysis of our phase-phonon data vs T, we first re-fitted the model curves for the Raman band data (Fig. 5(a,c) in main paper), i.e. the frequencies and bandwidths extracted from [3] (note that only the fitted values of the parameters κm_n^2 were given there). This yielded a fitted set of parameters as shown in Table I, where the values of κm_n^2 are in agreement with those given in [3].

1. Phenomenological impurity pinning/scattering vs. temperature

As discussed in the main paper, the nominal TDGL model summarized above predicts temperature-independent band parameters for the phase-phonons, whereas a clear T-dependence is observed in the experimental THz data (Fig. 5(b,d), main paper). Hence several physical generalizations/modifications of the model were investigated, in order to reconcile this T-dependence, which would still retain the good agreement with the literature amplitude-phonon data. This included (i) general damping (c.f. Eq. 3, i.e. including the underdamped regime); (ii) a temperature dependence for β – although this does not actually affect the T-dependence of the phonon data, rather only the magnitude of the equilibrium coordinates and renormalization of the critical temperature $T_{c0} \to T_c$. (iii) Our efforts to generalize the TDGL potential to include gradient terms of the complex order parameter (as suggested in [3]), in particular the term $|\partial_z\tilde{\Delta}|^2$ [7, 8], did not yield any coupling between the amplitude- and phase-channels (at least to first-order, once reducing the spatial equations to the $q = 2k_F$-components $\tilde{\Delta}$ and $\tilde{\xi}_n$). (iv) A finite pinning potential for the CDW phase [8–10], which was omitted in [3, 4] presumably as it should not strongly affect the amplitude channel and hence was not necessary to treat the behavior of the Raman-active bands. (v) T-dependent damping γ_2 for the EOP phase (Δ_2). In this case, one could consider two opposite trends: i.e. $\gamma_2(T)$ *increasing* with T, e.g. due to friction (scattering) with an increasing number of normal carriers thermally excited across the CDW gap [11]; $\gamma_2(T)$ *decreasing* with T, e.g. due to the loss of thermal carriers which may screen impurity/Coulomb interactions between charges in the CDW condensate and suppress the associated scattering processes [12, 13].

We found that including effects (iv) and (v) (with γ_2 decreasing with T) allowed one to reproduce the (semi-quantitative) behavior of the phase-phonon band data vs. T, i.e. with band A stiffening (and crossing its bare mode frequency $\nu_{0A}^{(0)}$ at intermediate T) while bands B,C soften as $T \to T_c$, and a significant reduction in the bandwidth of band A at low T. These effects were implemented as follows. For the impurity pinning, we treat a single strong-pinning center (which could be the consolidated average of several impurity potentials in each local region) and add to the function U (Eq. 2) a harmonic potential $U_i = \frac{1}{2}\Omega_i^2(T)\Delta_2^2$ [10, 14] (taking the origin of the impurity at $\varphi = 0$). As both impurity pinning and scattering should depend on the (equilibrium) amplitude of the EOP, we took both the restoring force $\Omega_i^2(T)$ and damping $\gamma_2(T)$ to depend on Δ_0, i.e. $\Omega_i^2(T) = \Omega_i^2(0)\delta_0^n(T)$ and $\gamma_2(T) = \gamma + \gamma_i(T)$ with $\gamma_i(T)/\gamma = g_i\delta_0^n(T)$, where $\delta_0(T) \equiv \Delta_0(T)/\Delta_0(0) = (1 - T/T_c)^{1/2}$ is the normalized EOP amplitude. Tests showed that the value $n = 2$ for the exponent was required to obtain reasonable agreement with the data (the physical significance of this is discussed in the main paper). The best fit of the phase-phonon data corresponded to the values $\Omega_i(0)/2\pi = 9.3$ THz and $g_i = 1.9$ (using the same values for the other parameters above, and hence main-

taining the fit to the literature amplitude-phonon data). The separate and combined effect of each term is illustrated in Fig. 3 (c.f. Fig. 5 in the main paper). In particular, one sees that the additional damping causes the eigenvalues to approach those of the bare modes as T decreases. This reduction in mixing between the (increasingly damped) EOP and the bare phonons can also be understood by considering the mechanical analogy of coupling of oscillators to an overdamped "bath". Also, the pinning potential is seen to play the dominant role for band A in terms the crossing of $\nu_{0A}^{(0)}$ at $T \approx 120$ K and the initial reduction in bandwidth with decreasing T.

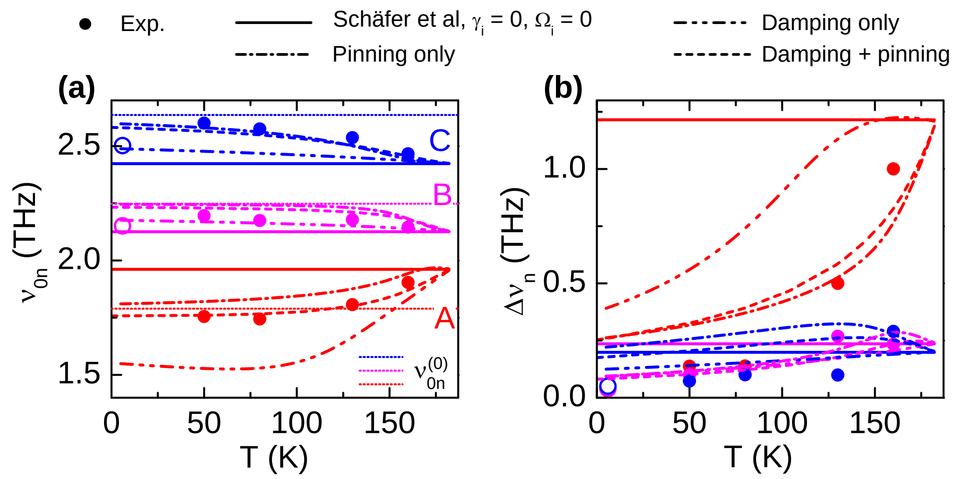

FIG. 3. Phase-phonons band parameters vs. T from experimental and TDGL model with impurity damping and pinning: (a) frequencies ν_{0n} and (b) bandwidths $\Delta\nu_n$. Description of the model parameters given in the text. To illustrate the impact of each effect separately, we plot the model curves in all four cases: no effects (the nominal TDGL model from [3, 4]), pinning only, damping only, and both pinning/damping (as indicated in legend).

2. *EOP-phonon coupling with both coherent and incoherent contributions*

As mentioned in the main text, the present results and those from the literature [15] indicate that only the phase-phonon bands are strongly affected (blue-shifted) by photoexcited free carriers. The issue was discussed in how to reconcile such a situation within the TDGL model. As asserted in the main paper, one explanation involves a loss of equivalence in the EOP-phonon coupling m_n for the amplitude and phase channels, due to the coupling losing a strict coherent dependence on the relative phases. Here we describe how this can be included in the TDGL model quantitatively.

The coupling term U_c between the EOP and each bare phonon is replaced by

$$U_c \rightarrow -m_n \Delta \cdot \xi_n [(1-\eta)\cos(\varphi-\chi)+\eta], \tag{5}$$

where $0 < \eta < 1$ represents the loss of coherence. Physically, this could arise in the photoexcited state due to local fluctuations, whereby the coexistence of the EOP and phonon amplitude still yields a stabilization of the local energy density, only that the strict local phase relation is suppressed. Using Eq. 5 in Eq. 2, one still obtains the same result for the equilibrium given above (Δ_0, ξ_{0n}). The Hessian about the equilibrium is

however altered, leading to the equations of motion:

$$\partial_t^2\hat{\Delta}_1 = -\left[2\alpha(T_c - T) + \frac{m_n^2}{\omega_{0n}^2}\right]\hat{\Delta}_1 + m_n\hat{\xi}_{n1} - \gamma_1\partial_t\hat{\Delta}_1 \tag{6a}$$

$$\partial_t^2\hat{\Delta}_2 = -\frac{m_n^2(1-\eta)}{\omega_{0n}^2}\hat{\Delta}_2 + m_n(1-\eta)\hat{\xi}_{n2} - \gamma_2\partial_t\hat{\Delta}_2 \tag{6b}$$

$$\partial_t^2\hat{\xi}_{n1} = m_n\hat{\Delta}_1 - \omega_{0n}^2\hat{\xi}_{n1} \tag{6c}$$

$$\partial_t^2\hat{\xi}_{n2} = m_n(1-\eta)\hat{\Delta}_2 - \omega_{0n}^2\hat{\xi}_{n2} \tag{6d}$$

Comparison with Eq.s 3 shows that the amplitude channel is unaffected (Eq.s 6(a,c)), while the effective coupling for the phase channel is reduced (essentially by a factor $(1-\eta)$, although the first term in Eq. 6(b) corresponds rather to $m_n \to m_n\sqrt{1-\eta}$). Hence, this modification predicts that the phase-phonons should be shifted toward their bare frequencies upon photoexcitation, as per the dominant trend seen in the data (see Fig. 5(b) in the main paper).

E. Comparison of the TDGL model with quantum mechanical models

It is instructive to compare the qualitative predictions of the TDGL model with those from earlier quantum-mechanical treatments (QM) in the literature (based on the Fröhlich Hamiltonian). The extension of the original LRA model [16] to $N>1$ bare phonons [17] provides a closed-form solution for the $Q=0$ amplitude- and phase-phonon spectra for $T \to 0$. This yields N renormalized modes for each channel, with $\nu_{0,n-1}^{(0)} < \nu_{0n} < \nu_{0n}^{(0)}$ (as well as the single-particle gap near 2Δ in the conductivity spectrum). For typical values of the e-ph coupling coefficients, one has: (i) the lowest amplitude mode ("amplitudon") is most strongly red-shifted; (ii) the lowest phase mode ("phason") is driven to $\nu = 0$ (or close to it, if pinning is included [17]) leaving only $N-1$ renormalized phase-phonons at finite frequency. This contrasts to the predictions of the TDGL model, where, as detailed above, one has N finite-frequency modes also for the phase channel in the overdamped limit (and an additional mode at $\nu = 0$). Hence in the nominal QM theory, we would have consider a re-assessment of our assignment for band A (the lowest-frequency phase-phonon), and, for that matter, the basis of the TDGL model. We note that subsequent extensions of the QM model to include long-range Coulomb terms [18, 19] predict that the phason resonance can actually break up into both acoustic and optical resonances, the latter of which could provide an alternate assignment for our band A (although the T-dependence of the example results in [18] do not correlate with the frequency/bandwidth behavior of our band here). Interestingly, if one reduces the phase damping γ_2 in the TDGL model to the underdamped regime, we find that one obtains a set of phase-phonons qualitatively consistent with the QM model (i.e. with an additional phase-mode at $\nu = 0$ and with non-zero damping), although this does not readily aid in interpreting our results here. Conversely, one could consider that the interactions included to calculate the QM spectral response functions do not adequately predict the phase damping, and should rather converge to the TDGL result if this were incorporated. However, in this case, the QM theory would no longer predict the phason at $\nu \sim 0$, where a band with a peak at $\nu \sim 100$ GHz has indeed been assigned in experimental studies [20]. Clearly this issue deserves further theoretical investigation, as we assert in the main paper.

F. Simulations of transient broadening of phonon response around delay zero

As presented in the main paper, some of the initial spectral features in the reflectivity spectra $\Delta r/r_0$ (i.e. for $\tau \lesssim 2$ ps) could not be fitted using the quasi-stationary Drude-Lorentz dispersion model, where a form of

transient broadening occurs for the Lorentzian bands. This phenomena is primarily due to the frequency-mixing which occurs when the THz probe pulse interacts with a system whose polarization response changes on the time scale of the THz cycle period. While these effects have been studied theoretically for a Drude response (e.g. [21]), we did not find any exposition for the perturbation of (narrow) Lorentzian bands, where the temporal response (free-induction decay) is longer and hence temporal-spectral effects can be more severe. Here we present the simulation results of such OP-TP reflection experiments, for the case of a single Lorentzian band which undergoes a blue-shift in its instantaneous response on a short time scale, to demonstrate how the experimentally observed broadening (as identified in the main paper) manifests.

While the finite-difference time-domain (FDTD) simulations are a proven method to simulate pump-probe experiments with rapidly changing response functions [22, 23], these can be time-consuming, especially for batch runs (i.e. vs. pump-probe delay τ). Instead, here we develop and apply a model for a homogeneously excited thin-film between air (refractive index n_1) and substrate (n_3) which allows time-integration of only a small number of field/polarization values. The treatment is based on applying the time-domain boundary conditions for the fields at both interfaces and expanding the field development to first-order in the sample depth ($z \in [0,L]$). The resulting coupled equations for the reflected (E_r) and transmitted (E_t) fields, in terms of the known incident field (E_i) are

$$\frac{\partial E_\mathrm{r}}{\partial t} = \frac{\partial E_\mathrm{i}}{\partial t} + \frac{c}{n_1 L}(-E_\mathrm{i} + E_\mathrm{r} + E_\mathrm{t}) \tag{7a}$$

$$\frac{\partial E_\mathrm{t}}{\partial t} + \frac{1}{\varepsilon_0}\frac{\partial}{\partial t}P\{E_\mathrm{t}\} = \frac{2n_1 c}{L}(E_\mathrm{i} - E_\mathrm{r}) - \frac{2n_3 c}{L}E_\mathrm{t} - \frac{\partial}{\partial t}\left[E_\mathrm{i} + \frac{1}{\varepsilon_0}P\{E_\mathrm{i}\}\right] - \frac{\partial}{\partial t}\left[E_\mathrm{r} + \frac{1}{\varepsilon_0}P\{E_\mathrm{r}\}\right] \tag{7b}$$

where each $P\{E_n\}$ is the co-integrated time-domain solution to the corresponding polarization equation

$$\frac{\partial^2 P\{E_n\}}{\partial t^2} + \Gamma(t)\frac{\partial P\{E_n\}}{\partial t} + \omega_0^2(t)P\{E_n\} = S(t)E_n(t) \tag{8}$$

where $\Gamma(t)$, $\omega_0(t)$ and $S(t)$ are the time-dependent damping, resonant frequency and strength of the electronic oscillator (whose temporal development is initiated by a pump pulse centered at $t = -\tau$). These equations are readily generalized to multiple Lorentzian bands and the addition of an instantaneous response due to ε_br. Their validity was checked by comparison of numerical data for a time-stationary medium with the analytic formula for the reflected/transmitted fields. To simulate the experimental data, Eq.s 7-8 are integrated for a large set of τ-values. The resulting raw data (integrated vs. laboratory time t_lab) are then interpolated onto the experimental time base t (which is skewed relative to t_lab due to the use of delay stage in the THz emitter beam path [24]), and after time-windowing and Fourier transformation $\Delta r(\nu,\tau)/r_0(\nu)$ is calculated.

As a representative simulation for comparison with the experimental 2D signals $\Delta r(\nu,\tau)/r_0(\nu)$ (see Fig. 3(c,d) in main paper), we consider the case of a single Lorentzian band, whose frequency and strength are perturbed by excitation with a Gaussian pulse with duration 150 fs, as shown in Fig. 4(a). Also shown for comparison is the "fictitious" simulated data $\Delta r/r_0$ obtained if one uses a stationary response for each "frozen" value of the Lorentzian band parameters (which would be the case for a much slower transition in the parameters). Clearly one observes the dynamic broadening artifact in the simulated data (especially during the first ~2 ps), which bears a close resemblance to those in the experimental data (both for the real and imaginary parts of $\Delta r/r_0$). Note that this is an inherent effect in light-matter interaction close to the uncertainty limit (i.e. during rapid evolution compared to the oscillation period, spectral resonances must become broadened), and accentuates that OP-TP transients on a sub-ps time scale cannot be interpreted directly in terms of a quasi-stationary picture. A more rigorous approach is to consider the response of the perturbed system rather in the dual-frequency domain [24], although this blurs the ability to analyse/interpret the time-evolution of the spectral response (which becomes well-defined again for longer time scales).

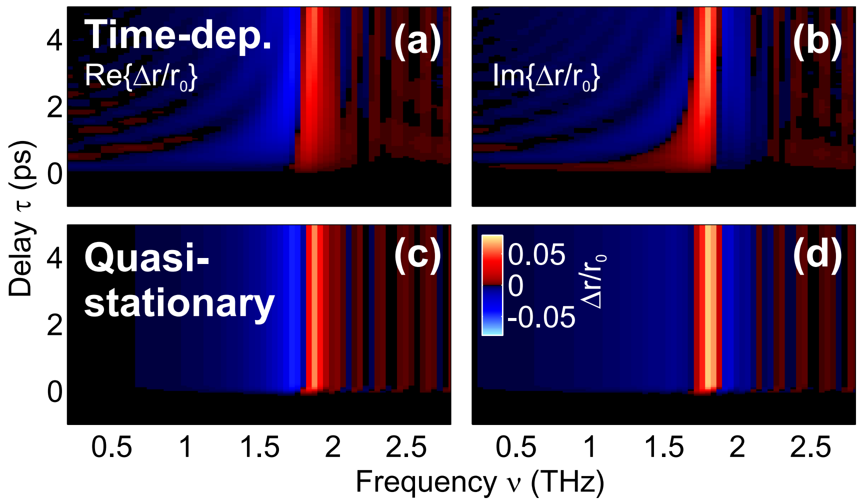

FIG. 4. (a) Simulation of differential reflectivity spectra $\Delta r(\nu,\tau)/r_0(\nu)$ vs. pump-probe delay τ, from integration of time-non-stationary polarization response in a thin layer with homogeneous excitation ($D_{\mathrm{ex}} = 2\ \mu\mathrm{m}^{\mu\mathrm{m}}$), for a model system where a ground-state phonon at $\nu = 1.75$ THz undergoes a photo-induced shift to 1.85 THz and weakens by 20% (pump pulse duration 150 fs). The substrate refractive index was taken as frequency-independent, $n_3 = \sqrt{80}$. (b) Corresponding idealized quasi-stationary response, to demonstrate how the model band parameters change with delay. The same temporal time windowing was employed in both cases before Fourier transformation, in order to best represent the experimental data in the main paper (Fig. 3(c-d)).

[1] J. Y. Vinet, M. Combescot, and C. Tanguy, Solid State Communications **51**, 171 (1984).
[2] F. Meng, M. D. Thomson, B. E. Sernelius, M. Jörger, and H. G. Roskos, Phys. Rev. B **91**, 075201 (2015).
[3] H. Schäfer, V. V. Kabanov, M. Beyer, K. Biljakovic, and J. Demsar, Phys. Rev. Lett. **105**, 066402 (2010).
[4] H. Schäfer, V. V. Kabanov, and J. Demsar, Phys. Rev. B **89**, 045106 (2014).
[5] R. N. Bhatt and W. L. McMillan, Phys. Rev. B **12**, 2042 (1975).
[6] D. Khomskii, *Basic aspects of the quantum theory of solids* (Cambridge University Press, New York, 2010).
[7] D. J. Scalapino, M. Sears, and R. A. Ferrell, Phys. Rev. B **6**, 3409 (1972).
[8] R. H. McKenzie, Phys. Rev. B **52**, 16428 (1995).
[9] J. R. Tucker, Phys. Rev. B **40**, 5447 (1989).
[10] W. Wonneberger, J. Phys. Condens. Mat. **11**, 2637 (1999).
[11] W. L. McMillan, Phys. Rev. B **12**, 1197 (1975).
[12] P. Bak, in *Electron-phonon interactions and phase transitions*, NATO advanced study insitututes series: Series B, Physics, Vol. 29, edited by T. Riste (Plenum Press, New York, 1977) Chap. Phase transitions in quasi one-dimensional metals (TTF-TCNQ and KCP), p. 66.
[13] P. Bak and S. A. Brazovsky, Phys. Rev. B **17**, 3154 (1978).
[14] S. Turgut and L. M. Falicov, Phys. Rev. B **50**, 8221 (1994).
[15] A. Tomeljak, H. Schäfer, D. Städter, M. Beyer, K. Biljakovic, and J. Demsar, Phys. Rev. Lett. **102**, 066404 (2009).
[16] P. A. Lee, T. M. Rice, and P. W. Anderson, Solid State Commun. **14**, 703 (1974).
[17] M. J. Rice, Solid State Commun. **25**, 1083 (1978).
[18] K. Y. M. Wong and S. Takada, Phys. Rev. B **36**, 5476 (1987).
[19] A. Virosztek and K. Maki, Phys. Rev. B **48**, 1368 (1993).
[20] L. Degiorgi, G. M. B.Alavi, and G. Grüner, Phys. Rev. B **44**, 7808 (1991).
[21] H.-K. Nienhuys and V. Sundström, Phys. Rev. B **71**, 235110 (2005).
[22] C. Larsen, D. G. Cooke, and P. U. Jepsen, J. Opt. Soc. Am. B **28**, 1308 (2011).
[23] M. D. Thomson, S. M. Tzanova, and H. G. Roskos, Phys. Rev. B **87**, 085203 (2013).
[24] H. Němec, F. Kadlec, and P. Kužel, J. Chem. Phys. **117**, 8454 (2002).
